# P-1443. Experiences with Human Immunodeficiency Virus (HIV), Dialysis, and Kidney Transplantation

**DOI:** 10.1093/ofid/ofae631.1615

**Published:** 2025-01-29

**Authors:** Meghna Nagam, Anne Huml, Anita R Modi

**Affiliations:** Cleveland Clinic, Cleveland, Ohio; Cleveland Clinic Foundation, Cleveland, Ohio; Cleveland Clinic Foundation, Cleveland, Ohio

## Abstract

**Background:**

People living with HIV and advanced kidney disease face unique challenges in the kidney transplant evaluation process. Retrospective chart reviews concerning this marginalized patient population portray kidney transplant candidates and the transplant evaluation process through the lens of the provider. We directly solicited patient perspectives to better understand their experiences with HIV, dialysis, and kidney transplantation.

**Methods:**

We recruited 20 people living with HIV and advanced kidney disease seen at our center to participate in one-on-one interviews guided by a structured questionnaire, audio-recorded, and professionally transcribed. Two physicians coded and reviewed all responses for common themes. A third physician served as the external thematic auditor. Data was collected on demographics, HIV care, and engagement in the transplant evaluation process for each participant.

**Results:**

All 20 participants (18 Male, 14 Black, Mean Age 54y) were prescribed integrase inhibitor-based regimens and 17 demonstrated suppressed HIV viremia. One was not referred for kidney transplantation, one referred but not yet evaluated, nine undergoing evaluation, three with referrals closed, one listed, and five transplanted. Twenty parent codes were identified, each with 1-10 sub-codes. Seven major themes arose: HIV-associated stigma inside and outside of healthcare; Balance of trust and autonomy in decisions to start therapy for HIV and advanced kidney disease; Effect of health literacy on HIV and advanced kidney disease treatment adherence; Communication failures during the transplant evaluation process; Health and lifestyle changes related to dialysis and transplant listing; Impact of social support on pre- and post-transplantation experiences; and Personal and medical consequences of the perception of the “ideal transplant candidate”.

**Conclusion:**

Patient perspectives offer valuable insight into the experiences of people living with HIV and advanced kidney disease undergoing dialysis and pursuing transplant evaluation. Attention to common attitudes and beliefs associated with this complex process, as well as its influence on life outside of healthcare, may guide future interventions to increase this vulnerable population’s access to kidney transplantation.

**Disclosures:**

**All Authors**: No reported disclosures

